# The interplay of exercise, immunity, and suboptimal health in college students: an integrative narrative review with public health implications

**DOI:** 10.3389/fpubh.2026.1996598

**Published:** 2026-10-29

**Authors:** Ruohan Li

**Affiliations:** College of Physical Education, Jilin University, Changchun, China

**Keywords:** chronic low-grade inflammation, college students, exercise immunology, psychological stress, public health, sedentary behavior, sleep, suboptimal health status

## Abstract

**Background:**

The immune system, vital for pathogen defense and tissue repair, is strongly influenced by lifestyle. With declining physical activity and rising chronic disease burdens, the role of exercise in modulating immunity has drawn growing attention. College students, undergoing critical life transitions, are susceptible to lifestyle-related risks such as sedentary behavior, insufficient sleep, and high psychological stress, which often lead to suboptimal health status.

**Population risks in college students:**

Immune and inflammatory risks are a concern among college students: prolonged sedentary behavior, chronic sleep deprivation, and elevated psychological stress frequently co-occur. Each of these behaviors is associated with low-grade inflammation and altered immune regulation in adults, and their clustering in students is therefore a reasonable basis for concern, although the strength of the association with immune outcomes has seldom been measured directly in this group. The term suboptimal health status is used in this review as a self-reported functional syndrome, defined in Section 3.4, rather than as an established clinical entity.

**Effects and mechanisms of different exercise behaviors:**

We examine the effects of different physical activities on immune function, chronic low-grade inflammation, and suboptimal health status in college students, outlining the basic framework of exercise immunology and distinguishing acute from chronic exercise responses. Evidence is categorized by exercise type, including moderate-intensity aerobic exercise, high-intensity training, resistance training, and mind–body practices, and their mechanisms, such as myokine-mediated anti-inflammatory signaling, immune cell mobilization, fat loss, and the mediation of sleep and emotion, are summarized.

**Methodological challenges:**

We critically discuss the methodological challenges in this field, separating two sources of disagreement that are often conflated: study design, which determines the causal question a study can answer, and measurement method, which determines how accurately exposure and outcome are captured. We analyze the limitations of each, including the inability of cross-sectional studies to establish causality, measurement bias in self-reported data, and ecological validity and compliance issues in trials, and we note how the same distinction applies to outcome assessment, from self-reported symptoms to immune-cell, cytokine, and clinical infection measures.

**Research gaps:**

Notable gaps are identified, including the “population mismatch” whereby findings from other populations are extrapolated to college students, limitations in sample selection and measurement standardization, and the lack of individualized and comprehensive intervention studies.

**Research significance and public health implications:**

This narrative review underscores the importance of understanding the relationship between exercise and immunity in college students, provides an overview of current knowledge, identifies critical limitations, and proposes future directions. Evidence was assembled through a structured narrative search, and findings obtained directly in college students are distinguished throughout from those extrapolated from athletic or clinical populations. It also offers public health implications for Chinese university settings, emphasizing targeted health education, campus-wide exercise interventions, sedentary behavior reduction, and integrated sleep and stress management to promote student wellbeing.

## 1 Introduction

The immune system is responsible for recognizing and eliminating pathogens and abnormal cells, and it also plays a role in repairing damaged tissues. It is influenced by genetics and shaped by daily lifestyle. Over the past century, physical activity levels in humans have declined markedly, while the burden of chronic diseases such as diabetes and cardiovascular disease has increased, prompting researchers to pay growing attention to the role of physical activity (1). The effect of exercise on immunity depends on volume and intensity: during a single bout of exercise, sympathetic nervous system activation and elevated adrenaline rapidly mobilize immune cells such as natural killer cells into the bloodstream, providing immediate immune surveillance (2). In contrast, long-term regular exercise improves immune function at baseline and reduces the chronic low-grade inflammation associated with aging and metabolic disorders (3). These two mechanisms underlie the saying that “exercise is medicine.”

This decline in physical activity has become a global phenomenon rather than a feature of affluent societies alone. Pooled analyses of population-based surveys across many countries indicate that more than a quarter of adults worldwide remain insufficiently active, and the global prevalence has changed little over recent decades (4). International guidelines recommend that adults accumulate 150 to 300 min of moderate-intensity aerobic activity each week, together with regular muscle-strengthening activity, and that they limit sedentary time, yet a large share of the population meets neither the activity nor the sitting recommendation (5). Young adults sit at the center of this transition. University students, in particular, spend a large part of the day seated: a meta-analysis of studies from multiple countries estimated an average of about 7 h of self-reported sitting per day, and considerably more when the same behavior is measured with accelerometers (6). The same period of life carries a heavy burden of psychological distress, with pooled estimates placing the prevalence of depressive and anxiety symptoms among college students at roughly one-third and two-fifths, respectively (7). Sleep is drawn into the same pattern, and a recent synthesis of sleep health in China frames insufficient and poor-quality sleep as a population-level public health challenge rather than as an individual failing (8). Taken together, these trends indicate that many students’ daily routines diverge from the behaviors that support immune health.

From another perspective, physical inactivity and prolonged sitting are independent health risks in their own right, rather than simply an absence of exercise. Long-term inactivity promotes the accumulation of visceral fat, and adipose tissue releases pro-inflammatory factors such as tumor necrosis factor-alpha (TNF-α) and interleukin-6 (IL-6), resulting in persistent, low-grade systemic inflammation. This, in turn, increases the risk of insulin resistance, atherosclerosis, and a range of chronic diseases (1, 9). Cohort studies have also found that prolonged sitting at work each day is associated with higher risks of all-cause and cardiovascular mortality, while increased physical activity during leisure time can partially offset this risk (10). Exercise therefore helps “balance” the body through immune and inflammatory pathways, offering benefits that extend far beyond weight loss or strength enhancement.

College students represent a particularly noteworthy group in this context. They are transitioning from adolescence to adulthood, with lifestyles that are not yet fully established and relatively easy to adjust. The reality, however, is less optimistic: they face considerable academic and job-seeking pressures, spend increasing amounts of time on screens and sitting, and generally suffer from insufficient sleep. Surveys in China provide ample evidence. A study involving over 20,000 students in Zhejiang Province found that about 40% exceeded 2 h of daily screen time, often accompanied by insufficient physical activity and poor sleep (11). Another survey among university students from four institutions in Anhui Province revealed that more than half were late-night sleepers, with most using their mobile phones for over 3 h daily, and that late sleep patterns correlated with poorer sleep quality (12). A questionnaire conducted during pandemic lockdowns at a university in Nanjing also indicated intertwined relationships among screen time, sleep patterns, and mental health among college students (13). From the perspective of 24-h activity guidelines, only a small proportion of college students meet recommended standards for physical activity, sedentary behavior, and sleep simultaneously, and compliance levels are associated with the risk of mood disorders (14). A survey at a comprehensive university in Hunan similarly showed that low levels of physical activity among students were linked to depressive tendencies (15).

The university years matter for public health because they fall within a transitional window in which adult habits are consolidated. Students leave the structured schedules of secondary school, gain control over their own time, and begin to make independent decisions about eating, sleeping, and moving, so the behaviors they form during this period tend to persist into later adulthood, when the costs of inactivity and chronic inflammation become far larger (6). College students are therefore both a high-risk group and a strategically important one for early intervention: the same years that allow harmful routines to take hold also allow them to be redirected before they harden. Because the lifestyle factors that shape long-term disease risk also act on the immune system in the near term, understanding how physical activity modulates immune balance and chronic low-grade inflammation in this group carries value beyond the explanation of biological mechanisms, informing preventive strategies that a university can realistically deliver.

Chronic psychological stress weakens cellular immunity and elevates inflammation through the hypothalamic–pituitary–adrenal axis and the sympathetic nervous system, and this evidence is already well established in young adults (16). Sedentary behavior, insufficient sleep, and high stress often coexist and exacerbate one another, pushing many college students toward suboptimal health status, a condition marked by mild immune imbalance and low-grade inflammation. Suboptimal health status refers to a functional discomfort state without clear organic disease, characterized by persistent fatigue, poor sleep, and reduced attention, and it is commonly assessed using standardized scales in China. Surveys of Chinese college students suggest that suboptimal health status is widespread in this group and that it tracks closely with psychological symptoms, indicating that the syndrome is a common feature of student life rather than a marginal concern (17). Physical activity interventions have repeatedly been shown to alleviate these symptoms, and systematic reviews support that regular exercise reduces the risk of poor sleep quality, anxiety, and stress among college students (18).

This narrative review focuses on college students and examines the effects of different physical activities on immune function, chronic low-grade inflammation, and suboptimal health status. It first outlines the basic framework of exercise immunology, then analyzes the immune and inflammatory risks among college students in light of their sedentary lifestyles, insufficient sleep, and high stress levels. The review subsequently organizes the evidence by type of exercise, summarizes the underlying mechanisms, compares the differences among cross-sectional studies, questionnaires, and experimental designs, and finally identifies current research limitations and promising directions for future study. Throughout, the underlying immunology serves as an explanatory tool that connects each mechanism back to the daily realities of campus life and to the practical question of how universities can promote student health.

### 1.1 Search strategy and selection criteria

This article is a narrative review rather than a systematic review, and it does not aim to quantify pooled effects. We searched PubMed/MEDLINE, Web of Science, and the China National Knowledge Infrastructure (CNKI), combining terms for physical activity and sedentary behavior (“exercise,” “physical activity,” “sedentary behavior,” “prolonged sitting”) with terms for immune and inflammatory outcomes (“immune function,” “salivary immunoglobulin A”, “natural killer cells”, “inflammation,” “C-reactive protein,” “interleukin-6”) and for the population or context (“college students”, “university students”, “young adults”, “suboptimal health status”, “sub-health”). The search covered database inception through the most recent update in 2026, and the last search was run in September 2026.

We considered systematic reviews and meta-analyses, consensus and position statements, randomized and non-randomized controlled trials, cohort and cross-sectional studies, and narrative reviews with a defined scope. Eligible records had to address physical activity, sedentary behavior, or exercise in relation to an immune, inflammatory, or suboptimal-health outcome in young adults or college students. We excluded animal-only studies, clinical populations and athletic cohorts used as though they represented general students, and records without a retrievable outcome measure, and we did not include grey literature. Reference lists of the retrieved reviews were screened for additional sources. The search was not intended to be exhaustive; it was designed to cover the main lines of evidence needed for each thematic section.

We prioritized studies conducted in college or university students whenever such evidence existed, and used evidence from healthy young adults, and then from general adult, older, clinical, or athletic populations, only where student-specific findings were absent or insufficient. Throughout the sections that follow, each statement indicates whether it rests on data collected in college students themselves (direct evidence), on evidence from other human populations (extrapolated evidence), or on a biological interpretation that has not been tested directly in students. Where studies disagreed, we report the disagreement rather than averaging it away, and we identify whether the divergence is more plausibly attributable to the population studied, to the study design, or to the method of measurement. Because the field is heterogeneous in design and measurement, the findings were synthesized narratively rather than pooled, and no formal risk-of-bias assessment was performed, a limitation acknowledged in the Discussion.

Generative AI was used to support drafting, literature organization, and language editing. It was not used to generate references or to interpret data. Every citation included in this review was checked against the original publication record, and the reference list reflects only sources we were able to verify directly.

## 2 Basic framework of exercise immunology

The biological framework outlined in this section serves an applied purpose, providing the terms needed to interpret why common student behaviors, such as a single gym session or years of inactivity, have different consequences for immune balance. To explain how exercise affects immunity, it is useful to distinguish between a single bout of exercise and long-term training. A moderate-to-high intensity exercise session can trigger dramatic changes in immune cells within minutes: sympathetic nervous system activation increases adrenaline and noradrenaline levels in the blood, mobilizing large numbers of natural killer cells, granulocytes, and other immune cells stored in the marginal pool and lymphoid organs into circulation, leading to a marked rise in their numbers for a period of time (2). After exercise ends, some of these cells redistribute back into tissues, causing their blood concentrations to decline. This process has sometimes been misinterpreted as “immunity being suppressed after exercise,” but it more accurately reflects the redeployment of immune cells throughout the body, a temporary enhancement of immune surveillance rather than a weakening of immune function (3).

A single bout of exercise can also disrupt inflammatory signaling. Intense exercise is accompanied by a transient, inflammation-like response that is essential for muscle repair and the development of training adaptation. Certain signaling molecules released during exercise, particularly IL-6 secreted by contracting skeletal muscle, exert anti-inflammatory effects both locally and systemically, inducing increases in anti-inflammatory factors such as interleukin-1 receptor antagonist (IL-1ra) and interleukin-10 (IL-10), while simultaneously suppressing the release of TNF-α (19). Within the same exercise session, inflammatory and anti-inflammatory signals are therefore coordinated in parallel, enabling the body to complete the cycle of damage, repair, and adaptation. It should be emphasized that the biological meaning of IL-6 is context-dependent: the transient IL-6 released from contracting muscle during exercise behaves differently from the chronically elevated IL-6 found in obesity, insulin resistance, and other inflammatory conditions, so IL-6 is better described as a signaling molecule whose effect depends on its source, timing, and duration than as simply pro-inflammatory or anti-inflammatory (19, 20).

The full benefits of regular exercise become apparent over time. Long-term engagement in moderate-intensity physical activity can reduce certain inflammatory markers at rest, which relates to exercise reducing visceral fat, improving metabolism, and “training” the hematopoietic system to produce fewer inflammatory white blood cells (21). Physical inactivity, by contrast, carries its own risk: prolonged sitting promotes fat accumulation and is associated with chronic low-grade inflammation, and this tendency toward inflammation even at rest forms the common ground for many chronic diseases (9).

Based on these insights, exercise immunology has long proposed a “J-shaped” dose–response relationship: as physical activity increases from none to moderate levels, the relative risk of upper respiratory tract infections initially decreases. Once the training load becomes excessive and recovery is insufficient, however, immune regulation may be disturbed and infection risk may rise again. This is the context in which the “open window” hypothesis was proposed, suggesting a period of relatively weakened immunity following strenuous exercise. Two clarifications are needed here, because the exposures involved are frequently treated as equivalent when they are not. First, a single bout of high-intensity exercise, prolonged endurance exercise, repeated strenuous training, insufficient recovery, functional overreaching, and frank overtraining syndrome are distinct conditions, differing in duration, cumulative load, and recovery, and the evidence for impaired immunity applies mainly to the later and more sustained of these rather than to a single demanding session. Second, the traditional interpretation of post-exercise immunosuppression has been challenged: several authors argue that the fall in circulating lymphocyte numbers during recovery largely reflects redistribution of cells into peripheral tissues rather than a genuine loss of immune competence, and that this redistribution may in fact enhance surveillance (3, 22). Contemporary work therefore describes the immune response to exercise as a temporary, coordinated redeployment of cells rather than a period of suppression, while still acknowledging that unusually heavy or prolonged training loads, particularly when recovery is inadequate, are associated with increased infection risk. The practical implication is that the dose, the duration, and the recovery interval, not intensity alone, determine the immunological outcome, and that simply stating that “exercise lowers immunity” or “exercise boosts immunity” is inaccurate.

For college students, this framework has direct implications. Their daily activities often fall into two extremes: almost no movement at all, or occasional bursts of intense exercise. The immune risks associated with these two extremes differ: the former involves long-term chronic low-grade inflammation and weakened immune surveillance, while the latter entails temporary disturbances after a single workout combined with inadequate recovery. With this dose–response framework in mind, the specific immune risks faced by college students can now be analyzed against a clear reference point.

## 3 Immune imbalance and low-grade inflammation risk in college students

### 3.1 Prolonged sitting and sedentary behavior

Sedentary behavior is one of the most common characteristics among contemporary college students. Long hours spent in classrooms, studying, and using computers and smartphones mean that a large portion of the day is spent sitting. The direct consequence of prolonged sitting is a drastic reduction in energy expenditure, which over time contributes to weight gain and increased visceral fat. Visceral fat is not merely inert storage tissue; it acts as an active inflammatory organ, in which macrophages within adipose tissue shift toward a pro-inflammatory phenotype and release factors such as TNF-α, placing the body into a state of low-grade inflammation even at rest (9). Although this condition does not manifest as acutely as an infection, it gradually and persistently disrupts immune system balance and lays the groundwork for future metabolic diseases. Cohort studies show that extended sitting is associated with increased mortality risk, while regular physical activity can partially counteract this effect (10). In China, surveys indicate that around 40% of students in Zhejiang Province spend excessive time on screens, with many college students filling their sedentary periods entirely with screen-based activities (11).

### 3.2 Insufficient sleep

Sleep deprivation among college students is common and familiar to students themselves. Staying up late to finish assignments, using smartphones before bed, and irregular sleep schedules have substantially reduced both the duration and the quality of sleep for many. A survey in universities in Anhui revealed that over half of the students are night owls, that most spend more than 3 h daily on their phones, and that late sleeping is associated with poorer sleep quality (12). Sleep is far more than “wasting time lying down”; it plays a crucial role in repairing the immune system, and reviews of the sleep-immune relationship describe sleep as a regulator of inflammatory signaling, T-cell function, and the response to vaccination (23, 24). Early experimental studies found that sleep deprivation increases the activity of pro-inflammatory factors: after partial sleep deprivation, healthy adults showed elevated levels of IL-6 and TNF-α secreted by monocytes upon waking the next morning, along with increased expression of related pro-inflammatory genes (25). Animal experiments further demonstrated that prolonged sleep deprivation can trigger a severe systemic inflammatory response (26). At the epidemiological level, genetically predicted insomnia has been linked to higher risks of infections such as sepsis, indicating that a causal relationship between sleep and infection resistance is plausible (27). For college students who already lack sufficient sleep, chronic sleep deficiency means that the body remains in a state of mild inflammation and weakened immune regulation for extended periods.

### 3.3 Psychological stress

College students face a highly concentrated set of stressors: academic coursework, examinations, grade point averages, graduate school preparation, and employment concerns, along with the adjustment to life away from home and to peer relationships. The impact of psychological stress on immunity is not merely metaphorical; it involves clear physiological pathways. Stress activates the hypothalamic–pituitary–adrenal axis and the sympathetic nervous system, leading to elevated levels of glucocorticoids and catecholamines. Although short-term stress may be beneficial, chronic long-term stress suppresses cellular immunity, for instance by weakening natural killer cell activity and altering lymphocyte responses (16), a pattern confirmed across three decades of studies in a meta-analysis that distinguished acute from chronic stressors (28). A classic example is that caregivers experiencing chronic stress from caring for older population dementia patients show weaker antibody responses to influenza vaccination, which correlates with elevated stress hormone levels (29). Direct evidence also exists among student populations: early studies found that academic stressors such as examinations are associated with reduced secretion rates of salivary secretory immunoglobulin A (s-IgA), a marker of diminished mucosal immunity (30). Students during examination periods may therefore experience a weakened immune defense. Direct evidence from this population also links higher perceived stress to a higher incidence of upper respiratory infection among undergraduates, although the cross-sectional design cannot establish the direction of that association (31).

### 3.4 Triple overlap and suboptimal health status

Sedentary behavior, insufficient sleep, and high stress rarely occur in isolation; instead, they often form a self-reinforcing cycle in which stress reduces the motivation to move and leads to later bedtimes, a lack of sleep results in poor energy the next day, and low energy increases screen time. Because the three behaviors cluster, it is reasonable to consider their combined rather than separate associations with health, and it is in this context that the concept of suboptimal health status is used.

The construct requires careful definition, because it is often treated as though it named a known condition. In the Chinese literature, suboptimal health status refers to a state without a clear organic disease diagnosis that is characterized by persistent fatigue, poor sleep, difficulty concentrating, frequent colds, and other functional discomforts. It is best understood as a symptom-based, preventive-health classification rather than a validated clinical entity: it is defined operationally by questionnaire responses, has no agreed pathological criterion, and its diagnosis rests on self-report. The instruments most widely used are the Suboptimal Health Status Questionnaire-25 (SHSQ-25) and its short form, developed and psychometrically evaluated within the predictive, preventive and personalized medicine framework, with item domains covering fatigue, sleep, digestive function, immune function, and mental status (32–35). The construct originated and remains predominantly used in Chinese and wider East Asian research, and although the instrument has been evaluated in a small number of non-Chinese populations, its validation specifically in university students is limited and much of the supporting psychometric work has been conducted in urban adult samples (34, 35). Its overlap with neighbouring constructs is substantial: the symptoms that define it, fatigue, psychological distress, sleep disturbance, and medically unexplained complaints, are also, and more conventionally, measured with established fatigue, depression, anxiety, and sleep instruments, and the two approaches identify broadly similar groups. This overlap is a reason for caution when the term is used, not a reason to discard it, since it captures a subclinical state that routine disease-oriented assessment tends to miss.

An equally important question is whether chronic low-grade inflammation has been demonstrated in people classified as having suboptimal health status, or whether it is principally a proposed mechanism. In our reading of the literature, the latter is closer to the case. Studies of suboptimal health status have documented its prevalence, its association with lifestyle factors and psychological symptoms, and, in some work, differences in routine immunological indices, but rigorous studies pairing SHSQ-based classification with standardized inflammatory biomarkers in the same individuals are scarce, and we found none in college students that also included an exercise intervention. The association between the syndrome and inflammation should therefore be presented as a plausible mechanism that remains to be tested rather than as an established fact.

Empirical associations and mechanistic hypotheses should be kept apart here, and this review does not assume a simple causal chain from sedentary behavior, sleep loss, and stress through inflammation to suboptimal health status. Lifestyle factors are consistently associated with the questionnaire score (36), and studies of Chinese college students report prevalences ranging from roughly one-fifth to more than half depending on the instrument and cutoff, with close tracking of the score and psychological symptoms (17, 37); these are associations in cross-sectional data. Nothing about them establishes that inflammation mediates the relationship, and reverse pathways, in which poor health reduces activity and disrupts sleep, are equally compatible with the observations. Where suboptimal health status is discussed in this review, it is therefore used as a summary label for three overlapping domains, mild immune dysregulation with chronic low-grade inflammation, a greater tendency toward common infections such as frequent colds, and psychological symptoms such as persistent fatigue and poor mood, with the understanding that the first of these is inferred more often than it is measured. For college students, one key question then arises: to what extent can different forms of physical activity help with this cluster of symptoms and, where it has been measured, with the underlying immune and inflammatory processes? This is the focus of the next section.

## 4 The impact of four different physical activities on immunity, inflammation, and suboptimal health status in college students

### 4.1 Moderate-intensity aerobic exercise

Moderate-intensity aerobic exercise, such as brisk walking, jogging, swimming, and cycling, is the most extensively studied and currently most recommended type of physical activity. Its benefits are evident on two levels. At the epidemiological level, higher aerobic fitness and regular physical activity are associated with a lower risk of respiratory infections, and individuals who consistently engage in moderate-intensity exercise tend to experience fewer upper respiratory tract infection episodes per year (38).

Mechanistically, regular aerobic exercise reduces resting inflammatory markers and helps maintain the responsiveness of immune cells, and meta-analyses of exercise training show reductions in circulating C-reactive protein across a range of adult populations (39). For college students, moderate-intensity exercise also offers practical advantages: it has low entry barriers, is easy to sustain, and supports energy recovery, making it particularly suitable for countering the chronic low-grade inflammation associated with sedentary lifestyles. Systematic reviews have also shown that regular exercise can reduce the risks of poor sleep quality, anxiety, and stress among college students (18), which in turn supports improved immune function, since sleep and emotional wellbeing are key regulators of immunity. Studies that measure immune endpoints directly in college students remain few and their results are mixed: salivary immunoglobulin A changed little after moderate exercise in college men, and in one cohort of undergraduates higher self-reported physical activity was not associated with fewer common-cold episodes (40, 41). Other student samples differ in humoral and cellular immune indices across activity levels, but these findings are small and cross-sectional (42). The scarcity of such studies is itself part of the problem this review addresses.

### 4.2 High-intensity exercise and the risk of overtraining

Another common type of physical activity among college students is sporadic, intense exercise, such as sudden sprinting, last-minute gym sessions pushing to exhaustion, or an abrupt increase in workout volume for weight loss. The immune consequences of such behaviors require careful consideration, and the first step is to keep distinct conditions apart. A single bout of high-intensity exercise, prolonged endurance exercise, repeated strenuous training, a state of insufficient recovery, functional overreaching, and overtraining syndrome are not equivalent exposures; they differ in duration, in cumulative load, and in whether recovery is adequate, and the evidence for impaired immune function applies mainly to the more sustained of these rather than to one demanding session. A single bout does reliably produce a marked mobilization of immune cells, chiefly natural killer cells, neutrophils, and monocytes, together with a smaller rise in lymphocytes, followed during recovery by a decline in circulating lymphocyte numbers as cells redistribute into peripheral tissues (2, 22). Whether that decline represents a period of genuinely weakened immunity remains debated. The traditional view, captured in the “open window” hypothesis, treats the hours after strenuous exercise as a window of reduced resistance to infection, whereas a contemporary reinterpretation holds that the fall in circulating cells largely reflects redistribution rather than immunosuppression, and that acute exercise should not be labelled immunosuppressive (3). Both readings agree that unusually heavy or prolonged training loads, especially when recovery is inadequate, are associated with more frequent upper respiratory tract infections and with slower recovery, so that fatigue and performance decrements persist between sessions (3, 22). For college students who are usually sedentary, sudden high-intensity exertion combined with academic stress and insufficient sleep may increase the likelihood that these consequences appear, though this has not been tested prospectively in this group. For such students, what is needed is not necessarily more intensity but gradual progression and sufficient recovery.

### 4.3 Resistance training and muscle factors

Resistance training, also known as strength training, is valuable for college students primarily for maintaining muscle mass and improving metabolic health. Its effects on the immune system are largely indirect, mediated through the concept of “muscle as an endocrine organ.” When muscles contract, they secrete a series of molecules called myokines, among which IL-6 has received the most attention. A common misconception should be clarified here: although IL-6 is often regarded as a pro-inflammatory factor, the IL-6 released by muscles during exercise exhibits anti-inflammatory properties. Human studies have shown that elevated plasma IL-6 levels induce increases in anti-inflammatory factors such as IL-1ra and IL-10 (20). This supports the core idea behind the “muscle as an endocrine organ” theory (19). Regular strength training can also improve body composition and reduce fat, thereby reducing low-grade inflammation at its source, and recent meta-analyses support this direction while indicating its limits: resistance training has been associated with small reductions in circulating C-reactive protein, with less consistent effects on IL-6 and TNF-α (43). Direct research linking resistance training to immune markers in college students remains limited, however, and most evidence is derived from mechanistic inference or studies conducted in adult or older populations, so the anti-inflammatory benefit of resistance training in students is a reasonable expectation rather than a demonstrated effect.

### 4.4 Mind–body exercise

In addition to purely physical activities, mind–body exercises such as yoga, tai chi, and Baduanjin have recently been included in discussions of exercise prescription. These practices are characterized by gentle movements and an emphasis on breath regulation and mental focus, and they clearly help alleviate stress. Since stress is a major contributor to immune imbalance among college students, mind–body exercises may exert their effects through an indirect pathway: reducing stress, improving sleep, and restoring immune balance. Controlled studies have shown that several weeks of mindfulness meditation training can enhance antibody responses in healthy adults following influenza vaccination, accompanied by changes in brain activity related to emotion (44). Systematic reviews also suggest associations between tai chi and qigong training and improvements in immune response and inflammatory markers (45). These low-intensity exercises are particularly suitable for students who rarely exercise or are chronically stressed. Randomized trials in college students, including a supervised twelve-week Baduanjin program, support the feasibility of these practices (46), and a recent network meta-analysis of exercise trials in university students indicates that structured programs can reduce symptoms of depression, anxiety, and stress (47). These practices therefore offer a realistic and feasible starting point for becoming more physically active.

### 4.5 Interrupting prolonged sitting with low-intensity activities

For many students, exercising for half an hour each day is already difficult, and a more realistic challenge may be how to break up long periods of continuous sitting throughout the day. The harms of prolonged sitting are, to some extent, independent of physical activity levels: even when weekly exercise targets are met, extended sedentary periods remain detrimental. The physiological basis for this lies in what happens to muscle during inactivity. Sustained sitting suppresses skeletal muscle lipoprotein lipase activity, so that uptake of circulating lipids and glucose falls (48, 49), and postprandial glucose and insulin responses are correspondingly higher after uninterrupted sitting. The loss of muscle contraction also reduces blood flow and shear stress in the leg arteries, transiently impairing endothelial function (50, 51). Interrupting sitting with brief stand-ups and short movement breaks is therefore itself a health-promoting behavior, even at low intensity, because it restores muscle contraction and vascular flow. Controlled studies support this: in a randomized crossover trial, breaking up prolonged sitting with two-minute bouts of light or moderate walking every 12 min lowered postprandial glucose and insulin relative to uninterrupted sitting (52), and short walking breaks have been shown to preserve endothelial function across a prolonged sitting period (50). For college students, this means getting up during class breaks, using standing desks for studying or working, and walking between classes, all of which interrupt the metabolic and vascular consequences of uninterrupted sitting. Much of this evidence still comes from adult populations, and direct interventions in university settings remain limited, so these strategies are best presented as promising and low-cost, with their effectiveness in students still to be confirmed.

### 4.6 Summary

Overall, different types of physical activity do not have equivalent effects on the immune system. Regular moderate-intensity aerobic exercise and mind–body practice are the types with the most consistent supporting evidence, although that evidence comes predominantly from adults and from students at the level of symptoms rather than immune markers. High-intensity training offers benefits in performance enhancement but requires careful control of volume, and its immunological risks are best understood as dose- and recovery-dependent rather than as a fixed property of intensity. Resistance training is expected to exert indirect anti-inflammatory effects through muscle-derived factors and reduced adiposity, a conclusion supported by meta-analyses in older adults but not yet demonstrated in students. Reducing sedentary behavior is best described as a low-cost and potentially scalable strategy rather than as the most cost-effective intervention, since it has not been compared economically with alternatives in this population. The central issue is that most conclusions about immune markers in college students are extrapolated from evidence obtained in adults or active populations. The few studies that measure immune and inflammatory outcomes directly in college students are small, usually drawn from a single campus, and mixed in their findings (40–42). This means the structural limitations of the available evidence must be acknowledged first when discussing differences across studies. Table 1 summarizes the representative evidence discussed in this review by evidence domain, population, exercise or exposure, outcome, study design, and main finding, and states for each entry whether it is directly applicable to college students.

**Table 1:** Summary of representative evidence, by evidence domain, population, exercise or exposure, outcome, study design, main finding, and direct applicability to college students.

Evidence domain	Population	Exercise or exposure	Outcome	Study design	Main finding	Directly applicable to college students?
Exercise immunology; immune-suppression debate (3)	Athletes and general adults	Acute and chronic exercise	Immune function, infection risk	Narrative review and conceptual reinterpretation	The fall in circulating lymphocytes after exercise largely reflects redistribution rather than a genuine decline in immune competence	No; extrapolated from athletes and adults
Exercise and infection risk (38)	Adults	Moderate aerobic fitness and activity	Upper respiratory tract infection	Observational	Higher fitness and regular activity are associated with fewer upper respiratory tract infection episodes	No; extrapolated from adults
Exercise immunology; mucosal immunity (40)	Adults	Exercise intensity and duration	Salivary immunoglobulin A	Experimental	Salivary immunoglobulin A changed little with exercise intensity and duration	No; extrapolated from adults, mixed findings
Physical activity and infection in students (41)	Undergraduates	Self-reported physical activity	Common cold episodes	Observational cohort	Higher self-reported activity was not associated with fewer common-cold episodes	Yes; direct student evidence, no association found
Exercise immunology; immune indices in students (42)	Students	Level of physical activity	Humoral and cellular immune indices	Cross-sectional comparison	Immune indices differed across activity levels, but samples were small and the design was cross-sectional	Yes; direct student evidence, small and mixed
Psychological stress and immunity (31)	Undergraduates	Social stress	Upper respiratory infection incidence	Cross-sectional	Higher perceived stress was associated with higher upper respiratory infection incidence	Yes; direct student evidence, direction not established
Academic stress and mucosal immunity (30)	Students	Academic examination stress	Salivary secretory immunoglobulin A	Observational across examination periods	Examination stress was associated with reduced salivary secretory immunoglobulin A secretion	Yes; direct student evidence
24-h movement behavior (14)	Chinese university students	Adherence to 24-h movement guidelines	Mood disorders	Cross-sectional	Few students met activity, sedentary, and sleep recommendations simultaneously, and compliance was associated with mood-disorder risk	Yes; direct student evidence
Sleep and circadian rhythm (12)	Chinese college students	Chronotype	Sleep quality	Cross-sectional	Later chronotype and late-night phone use were associated with poorer sleep quality	Yes; direct student evidence
Sedentary behavior and screen time (11)	Chinese students (Zhejiang)	Screen time	Physical activity and sleep	Cross-sectional	Around 40% exceeded 2 h of daily screen time, often with insufficient activity and poor sleep	Yes; direct student evidence
Sedentary behavior and mental health (53)	College students	Screen-based sedentary behavior	Anxiety	Cross-sectional	Screen-based sedentary behavior, but not total sedentary time, was associated with anxiety	Yes; direct student evidence
Suboptimal health status; lifestyle (37)	Chinese students	Lifestyle factors	Suboptimal health status	Cross-sectional	Lifestyle factors were associated with suboptimal health status	Yes; direct student evidence
Suboptimal health status; psychological symptoms (17)	Chinese college students	Suboptimal health status	Psychological symptoms	Cross-sectional	Suboptimal health status was widespread and tracked closely with psychological symptoms	Yes; direct student evidence
Mind–body exercise intervention (46)	College students	Supervised twelve-week Baduanjin program	Health-related physical fitness	Randomized controlled trial	The program was feasible and improved health-related physical fitness	Yes; direct student evidence, but fitness rather than immune outcomes
Exercise and mental health (47)	University students	Exercise modalities	Depression, anxiety, and stress	Network meta-analysis of randomized controlled trials	Structured exercise programs reduced symptoms of depression, anxiety, and stress	Yes; direct synthesis, but behavioral outcomes
Sedentary behavior; metabolic effects (52)	Adults	Breaking up prolonged sitting	Postprandial glucose and insulin	Randomized crossover trial	Short walking breaks lowered postprandial glucose and insulin relative to uninterrupted sitting	No; extrapolated from adults

## 5 Mechanism integration: how exercise influences immune balance

When the categorized evidence above is brought together, the effects of exercise on immunity and inflammation in college students can be summarized as several interconnected yet distinct pathways. These pathways operate on different timescales and act at different points in the inflammatory process, and it is their interaction, rather than any one of them alone, that accounts for the broad effect of exercise on health. Understanding how they connect also explains why the benefits of exercise are often characterized by an overall improvement rather than a change in a single parameter. Figure 1 summarizes these pathways and distinguishes those supported by direct human evidence from those that remain extrapolated.

**Figure 1:**
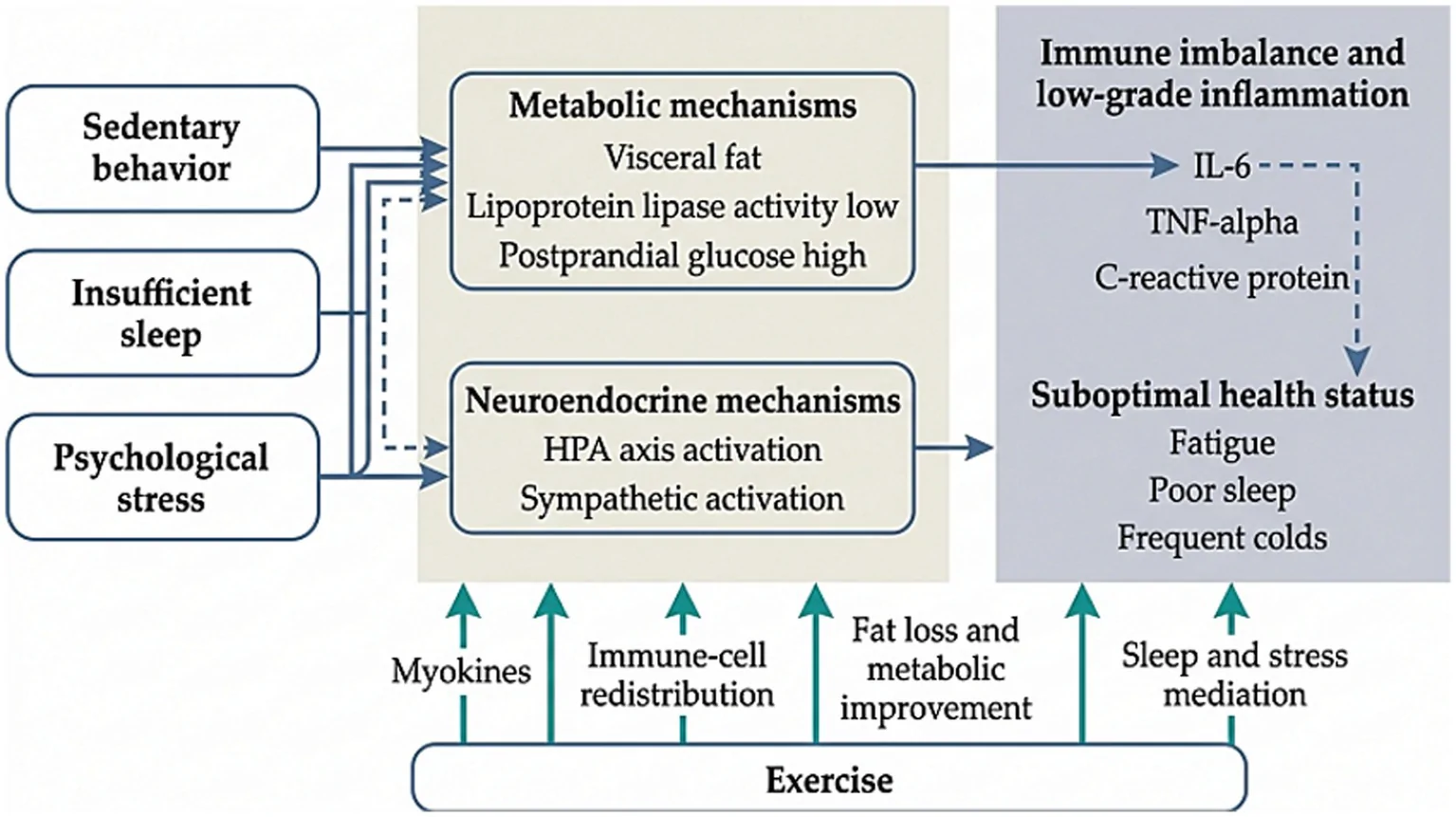
Conceptual pathways linking sedentary behavior, insufficient sleep, and psychological stress to immune imbalance, low-grade inflammation, and suboptimal health status, and the proposed action of exercise. Solid arrows denote pathways supported by direct human evidence; dashed arrows denote hypothesized or extrapolated pathways that have not yet been tested in college students.

### 5.1 From muscle to immunity: myokines and anti-inflammatory signaling

The most fundamental pathway involves molecular signals released by muscle contraction itself. During exercise, skeletal muscles secrete IL-6, which induces anti-inflammatory factors such as IL-1ra and IL-10 while simultaneously suppressing the release of TNF-α (19, 20). Exercise therefore creates a systemic inflammatory environment that tends toward “cooling down,” and with repeated long-term exposure the body’s baseline level of inflammation gradually decreases. This mechanism is particularly relevant for college students who are often on the verge of chronic low-grade inflammation: regular training lowers the body’s inflammatory baseline over time, whereas isolated bouts suppress inflammation only temporarily. Relevant reviews summarize this mechanism as the anti-inflammatory effect of exercise (54). Because this pathway works through circulating molecules and accumulates with repetition, its effects emerge over weeks of consistent training rather than within a single session, which distinguishes it from the immediate, cell-level responses described next.

### 5.2 Mobilization and redistribution of immune cells

The second pathway operates on a much shorter timescale, unfolding within a single session rather than accumulating across weeks. Sympathetic activation and catecholamines trigger the release of large numbers of immune cells such as natural killer cells into circulation, which then redistribute to peripheral tissues, forming repeated “patrols” (2). Regular physical activity also enhances the recirculation efficiency of immune cells. The significance of this mechanism lies in improved immune surveillance, which enables the immune system to detect and eliminate abnormal cells and potential threats more promptly. For students who are chronically sedentary, regular exercise can help restore a more responsive surveillance system. This pathway and the myokine pathway are complementary: mobilization puts immune cells where they are needed, while myokine signaling shapes the environment they encounter. Redistribution alone, however, does nothing to reduce the tissue sources of inflammation, and the pathway that addresses those sources operates over a longer horizon still.

### 5.3 Fat loss and metabolic improvement

The third pathway acts on the source of chronic inflammation rather than on the cells that respond to it, and its effects take the longest to appear. Over months, exercise reduces inflammation by controlling body weight and decreasing visceral fat. Visceral fat is a major source of low-grade inflammation, and exercise, particularly a combination of aerobic and resistance training, can reduce this harmful fat (1, 9). Exercise can also “train” the hematopoietic system to produce fewer pro-inflammatory white blood cells, thereby reducing the inflammatory burden on tissues such as the cardiovascular system at its source (21). This pathway explains why the anti-inflammatory effects of exercise become fully apparent only after several weeks or months of consistent activity. Its consequences then feed back into the other pathways, because a smaller visceral fat mass lowers the pro-inflammatory signal that immune cells encounter at rest, making the benefits of mobilization and myokine signaling easier to sustain. For a student whose sedentary routine has gradually increased visceral fat, this pathway responds more slowly than the others and depends most on sustained change, which is why campus programs that support months of consistent activity matter more than one-off events.

### 5.4 Through the mediation of sleep and emotion

The fourth pathway is indirect and particularly important for college students, because it works through two behavioral systems that are commonly disturbed in this group: sleep and emotional wellbeing. Exercise improves sleep quality and alleviates anxiety and stress (18), while adequate sleep and low stress levels in turn help maintain immune system balance and reduce inflammation (16, 25). Here the biological and behavioral pathways meet, since a student who sleeps well and feels less stressed is also more likely to keep exercising, and the effects reinforce one another over time. For students caught in the cycle of high stress, poor sleep, and reduced physical activity, exercise is among the most controllable starting points, precisely because it acts on the behavioral conditions that the other pathways depend on.

### 5.5 Mechanism convergence

These pathways are not independent but rather work synergistically, and their interaction is what gives exercise its broad effect. A moderate-intensity session activates the myokine-mediated anti-inflammatory response and mobilizes immune cells at the same time; repeated over weeks, the same behavior lowers visceral fat, improves sleep, and eases stress, so that the conditions sustaining low-grade inflammation are progressively removed. The pathways also reinforce one another in the reverse direction. Better sleep and lower stress reduce the pro-inflammatory drive that would otherwise blunt the benefit of exercise, and a smaller visceral fat mass lowers the baseline against which each new bout acts. This interdependence carries two implications. First, it explains why exercise is described as a regulator of “multi-organ dialogue”: its value lies in restoring coordination among the body’s systems rather than in changing a single parameter, and an intervention that acts on only one pathway cannot reproduce the combined effect. Second, it makes the effects of exercise difficult to capture with any single metric. Studies that report one immune marker, one inflammatory cytokine, or one behavioral outcome inevitably describe only part of what is happening, and the choice of which metric to measure can determine whether a study appears to show a benefit. This measurement problem is one important reason why the literature on exercise and immunity in college students appears more contradictory than the underlying biology would suggest. For campus health promotion, the convergence of these pathways adds a further implication: programs that address activity, sitting, sleep, and stress together are likely to be more effective than those targeting a single behavior, because each pathway sustains the others. These two consequences of interdependence lead into the methodological questions discussed in the next section.

## 6 Research designs and measurement methods: why findings appear to conflict

Reading the literature on exercise and immunity in college students reveals a puzzling phenomenon: for the same research question, different studies often yield inconsistent or even contradictory conclusions. Several distinct sources of disagreement are often conflated. The first is study design, which determines the causal question a study can answer, from cross-sectional observation at a single time point to experimental manipulation that can support causal inference. The second is exposure measurement, which determines how accurately physical activity and sedentary behavior are captured: self-reported questionnaires and device-based measures such as accelerometers can describe the same students differently. The third is outcome assessment, which determines what is actually being claimed, since self-reported symptoms, immune-cell measures, circulating inflammatory markers, and clinical infection outcomes are not interchangeable. The subsections below treat these axes separately before showing how they combine, since a study’s conclusion depends on the design it uses, the instruments it chooses for exposure, and the level at which it measures the outcome.

### 6.1 Cross-sectional designs: observing associations without causal inference

Cross-sectional studies measure physical activity habits and health indicators at a single point in time, most commonly revealing correlations such as “the more exercise, the better the immune and inflammatory markers.” Their advantages include large sample sizes, low cost, and the ability to quickly assess current conditions. Many of the surveys on Chinese college students mentioned earlier (screen time, sleep patterns, lifestyle) fall into this category (11, 12, 15). The central difficulty lies in the timing of measurement: because exposure and outcome are recorded simultaneously, cross-sectional designs cannot establish causality. Does exercise improve immunity, or are individuals who are already healthier and more energetic simply more inclined to exercise? The reverse effect, whereby healthier individuals tend to be more physically active, is likely real and may lead to overestimation of effects or even a reversal of the observed direction.

Two further features of the design generate additional bias, and together they explain much of the disagreement between otherwise comparable studies. The first is confounding by the cluster of behaviors that travel together. A student who exercises regularly is also more likely to sleep adequately, carry a lower screen-time burden, and report less stress, so a raw association between exercise and a favorable immune marker may partly reflect these companions rather than exercise itself. Analyses rarely adjust for all of them, and when adjustment is thorough the association often shrinks, sometimes to the point of disappearing. The second is selection. Students who volunteer for campus surveys, or who attend the lectures where a questionnaire is distributed, may differ systematically from those who do not, and the students at highest risk are often the least likely to take part. The practical consequence is that a cross-sectional study of exercise and immunity among college students can report a positive, null, or even negative association depending on which confounders were measured, which behaviors were adjusted for, and who happened to participate. Two studies with the same nominal question can therefore reach opposite conclusions without either being careless.

These limitations define what cross-sectional evidence can usefully contribute. Its value lies in description and hypothesis generation: it establishes how common inactivity, poor sleep, and low-grade inflammation are in a defined population, identifies the subgroups in which they cluster, and sets the stage for the questions that experiments later test. The error is not in using such studies but in reading a cross-sectional association as though it carried the causal weight of an intervention.

### 6.2 Experimental studies: causal inference with ecological validity and compliance bottlenecks

Experimental research, particularly randomized controlled trials, is the “gold standard” for causal evidence, because it can control confounding factors and establish causality. The causal inference power of randomized controlled designs far surpasses that of cross-sectional studies (55). Such designs, however, also face a series of practical challenges: sample sizes are often small, especially when blood tests for immune markers are involved; intervention durations are typically short, making it difficult to capture anti-inflammatory effects that emerge only after long-term training; and in real school settings it is hard to ensure that one group of students strictly follows an exercise regimen while another group does not exercise at all, so compliance problems and contamination dilute the observed effects.

The methodological challenges of trials in this setting run deeper than small numbers. First, a randomized trial buys internal validity at the cost of external validity: to make a protocol feasible, recruitment is often limited to a single campus and to students willing and able to attend supervised sessions, so participants tend to be fitter and better organized than the student body as a whole, and the resulting estimate may not transfer to the students who most need intervention. Second, the outcome itself is time-sensitive. Immune and inflammatory markers fluctuate with circadian rhythm, recent food intake, sleep, and the timing of the last exercise bout, so a single resting blood draw taken at an unstandardized moment introduces variability that can swamp a modest intervention effect; studies that fail to control the sampling window may miss a real effect or produce a spurious one. Third, an open-label exercise trial cannot blind participants to their allocation, and expectations can influence self-reported outcomes such as fatigue, sleep quality, and perceived stress, which are precisely the outcomes central to suboptimal health status. Fourth, the effective dose is difficult to fix. Compliance declines over weeks, incidental activity outside the program differs between groups, and contamination occurs when control-group students exercise on their own or train alongside the intervention group. Each of these problems throttles the signal the trial is designed to detect, and their combination explains why even well-conducted trials in this field often report modest and inconsistent effects.

The consequence for the study of college students is a paradox. The design best suited to answering the causal question is the least feasible to run at the scale and duration the question demands, so the field fills the gap with designs that are easy to run but cannot answer it. High-quality experimental evidence on immune and inflammatory outcomes in college students therefore remains scarce, and many of the conclusions about this group presented in earlier sections have had to rely on extrapolation from adult or athlete-based evidence, a limitation this review acknowledges.

### 6.3 Measurement methods: exposure and outcome assessment

Questionnaire-based studies are used almost exclusively in cross-sectional and most cohort studies to assess physical activity, with commonly employed tools such as the International Physical Activity Questionnaire. Physical activity is self-reported through recall, and this makes the measurement susceptible to memory bias and social desirability bias: people tend to overestimate their activity levels and underestimate sedentary time (56). Objective measurements can substantially alter conclusions: a study comparing accelerometer data with questionnaire responses found that accelerometer-measured physical activity showed stronger and cleaner associations with health indicators, indicating that self-reporting introduces considerable noise (57), and device-based studies in college students likewise produce estimates of sedentary time that differ from self-report and reveal associations with psychological outcomes that questionnaire data alone would miss (53). For college students, brief, low-intensity activities such as walking during class breaks or commuting on foot are particularly prone to being overlooked or misestimated by questionnaires, leading to inaccurate classifications of insufficient physical activity and sedentary behavior.

Measurement bias of this kind is systematic rather than random, and it distorts the picture in a direction that is hard to predict. Overestimation of activity compresses the range of exposure across a sample, so that genuinely inactive students are reclassified as moderately active; the effect is to attenuate any true association and to make exercise appear less protective than it is. Underestimation of sitting does the opposite, masking the contribution of sedentary time and shifting apparent risk onto other behaviors. Because questionnaires and device-based measures disagree systematically, two studies of the same students can reach different conclusions without either being careless: a questionnaire study may find that exercise is unrelated to an inflammatory marker, while an accelerometer study of the same sample detects a clear association. The discrepancy in that case is a property of the instrument rather than of the biology. This is why meta-analyses that pool questionnaire-based studies can dilute the very effects that device-based studies detect, and why the field increasingly treats self-report and objective measurement as complementary sources of evidence rather than interchangeable ones, using questionnaires to capture context, barriers, and subjective experience while reserving device-based measures for the exposure itself.

The same reasoning applies to how outcomes are assessed, an axis that is often collapsed into the design label. Studies in this field measure at least four different kinds of outcome, and a change in one does not imply a change in another. At the most subjective level are self-reported symptoms and questionnaire scales, including the instruments used to define suboptimal health status; these capture how students feel but are open to expectation and recall. Next are immune-cell measures, such as leukocyte counts and subsets, natural killer cell activity, and salivary immunoglobulin A, which describe the cellular and mucosal compartments but fluctuate with circadian rhythm, recent exercise, and stress. Then come circulating cytokines and systemic inflammatory markers, chiefly IL-6, TNF-α, and C-reactive protein, which are the most commonly reported biochemical endpoints and the most sensitive to the timing and standardization of sampling. Finally, there are clinical outcomes such as infection incidence and vaccine responses, which integrate the whole system over time and are the most meaningful to students but the hardest to measure and the least frequently reported. A study that finds a change in a circulating cytokine has not thereby demonstrated a change in infection risk, and a study that finds no change in a single resting marker has not shown that immune function is unaffected. Distinguishing outcome levels in this way clarifies why two studies of the same question can reach different conclusions while both being internally sound.

### 6.4 From opposition to complementation: integrating designs and measurement

These designs and measurement approaches form a single inferential sequence, each supplying what the next requires, and the contradictions between them are often a sign that the sequence is being read one link at a time. A sound approach would be to first identify issues and map distributions using cross-sectional studies and questionnaires in large samples; to then correct self-report bias with objective measurements such as accelerometers and wearable devices; and finally to test causality through rigorously designed randomized controlled trials with sufficiently large sample sizes and adequate follow-up.

The order matters because each design answers a question that the others cannot, and it supplies the assumptions the next stage depends on. Large cross-sectional and questionnaire studies establish prevalence and identify candidate associations, but they cannot order cause and effect. Objective measurement studies can be embedded within the same samples to repair the exposure variable, converting a noisy self-reported association into a cleaner one and revealing whether an apparent result was an artifact of misclassification. Only then does it make sense to commit the resources of a trial to testing the association that survived that correction. Conducted in this sequence, the designs inform one another: a trial launched on the basis of an unadjusted questionnaire association may spend years and scarce funding testing a relationship that better measurement would have dissolved, while a questionnaire study carried out without any intention of feeding into a trial produces correlations that no one can act on. Read this way, the disagreements between designs become diagnostic. A persistent divergence between self-report and device-based findings points to measurement as the problem, whereas a divergence between cross-sectional and experimental findings points to confounding or reverse causation. The contradiction identifies the weak link rather than undermining the field.

Translated into a concrete program for research on exercise and immunity in college students, this integration implies several specifications. Exposure should be measured objectively, ideally with accelerometers or wearables capable of separating sitting, light activity, and moderate-to-vigorous activity, while questionnaires are retained to capture context and subjective experience rather than to serve as the sole measure. Baseline immune and inflammatory markers should be drawn under standardized conditions at a fixed time of day, with food intake, sleep on the preceding night, and time since the last exercise bout recorded so that their contribution can be modeled instead of ignored. Because sleep and mood carry much of the behavioral pathway, they should be measured as intervening variables at multiple points rather than treated as nuisance covariates. Follow-up should be long enough for the biological endpoints of interest to respond, which for resting inflammatory markers means weeks to months rather than days. Finally, where a trial is infeasible, quasi-experimental designs such as stepped-wedge studies or natural experiments across campuses can preserve some causal leverage at population scale, provided their own assumptions are made explicit and tested. Taken together, these specifications describe a design in which longitudinal or intervention studies combine objective activity monitoring with standardized measurement of immune and inflammatory markers while incorporating sleep and mood as mediators.

Reading the literature as a sequence of linked designs, rather than as a competition among incompatible methods, converts its apparent contradictions into a roadmap for the studies that are still missing.

## 7 Discussion

This review set out to ask how different forms of physical activity shape immune function, chronic low-grade inflammation, and suboptimal health status in college students, and what the available evidence can and cannot support. Three conclusions stand out. First, exercise acts on immune balance through several pathways that operate on different timescales, from the immediate mobilization of immune cells during a single session to the slower reduction of visceral fat and improvement of sleep and mood that accumulate over months. Second, the type of activity matters as much as its amount: regular moderate-intensity aerobic exercise and mind–body practice are the most reliably supported, resistance training works mainly through muscle-derived anti-inflammatory signaling, high-intensity training carries dose-dependent risks that matter most for students who are sedentary for much of the week, and interrupting prolonged sitting is a distinct and largely additive behavior. Third, and most important for how the evidence should be read, the field is built largely on studies of adults and athletes, whereas the studies conducted directly in college students are few, small, and mixed in outcome (40–42).

Positioning these findings against the wider literature clarifies where the review adds value and where it must remain cautious. The mechanistic pathways described here are well established in exercise immunology, and the anti-inflammatory role of muscle-derived IL-6, the redistribution of immune cells after exercise, and the contribution of visceral fat to low-grade inflammation are supported by consistent adult and animal evidence (2, 19, 21). The same is true of the effect of prolonged sitting on muscle lipoprotein lipase activity, postprandial glycemia, and vascular function, which rests on controlled experiments in adults (48–50, 52). What is much less secure is the translation of these mechanisms to a general student population. Reviews of physical activity promotion in universities show that interventions can change student behavior, but they rarely measure immune or inflammatory outcomes, so the behavioral and the biological literatures remain largely separate (58, 59). The concept of suboptimal health status, likewise, is well operationalized as a self-reported syndrome in Chinese populations (32–35), yet only a small number of studies have applied it to college students, and fewer still have paired it with objective immune measures (17, 37). The review therefore connects two strands of evidence, mechanisms from exercise immunology and student-level epidemiology, without claiming that the connection has been directly tested.

The strength of the causal language used throughout reflects this hierarchy. Evidence from randomized trials and from controlled mechanistic experiments supports statements that a given behavior changes a measurable physiological parameter under specific conditions. Evidence from cross-sectional surveys supports statements about association and distribution rather than about cause. Where this review describes what exercise does for students, that description is a plausible extension of mechanisms demonstrated in other groups, and it is best read as a hypothesis to be tested in students rather than as an established effect. This distinction has practical consequences: a recommendation to increase moderate-intensity activity rests on the general evidence, whereas a specific prescription for immune benefit in students, such as a particular intensity or weekly volume, is not yet supported by data from this population.

A further consideration is that the effects of exercise on immune and inflammatory outcomes differ markedly across individuals, and the review has so far treated college students as a more homogeneous group than they are. Sex is one of the best-documented modifiers of immune function: women and men differ in immune cell composition, in inflammatory signaling, and in the risk of autoimmune and infectious outcomes, and these differences are relevant to any measurement of circulating markers (60, 61). Baseline fitness and adiposity shape both the exposure and the outcome, since a given session is a different physiological stimulus for a trained and an untrained student, and body composition influences resting inflammation. Behavioral and contextual factors add further variation: smoking and alcohol use, nutritional status, sleep duration, psychological stress, and, in women, hormonal factors including the menstrual cycle, can each shift the inflammatory markers being measured. Infection and vaccination status alter the same endpoints, and both physical activity and inflammation vary with season and with the timing of examinations. Most studies in this field report few of these variables and adjust for fewer of them, so the same intervention can appear effective in one sample and ineffective in another without any inconsistency in the underlying biology. A more informative reporting standard would describe the sample in these terms and, where feasible, test whether the effect differs by sex, fitness, or adiposity rather than reporting a single average for all students.

Several limitations of this review should be acknowledged. It is a narrative rather than a systematic review, and although a search strategy was defined (Section 1.1), study selection was interpretive and no formal risk-of-bias assessment was performed. Its focus on Chinese university settings reflects both the epidemiology of suboptimal health status and the availability of student surveys, which limits generalization to other systems. The mechanistic sections draw on studies that differ widely in design, sample, and outcome, so the synthesis is qualitative, and publication and language bias may have shaped which exercises and outcomes appear best supported, since many Chinese-language studies of student immune markers are not indexed internationally. These limitations define the scope of the argument rather than weaken it: the biological rationale for exercise as a regulator of immune balance is strong, the behavioral case for promoting activity and reducing sitting among students is sound, and the missing link is direct evidence in students themselves. That gap shapes both the research agenda and the caution with which the practical implications that follow are offered.

## 8 Research limitations and future trends

Summarizing the above points, current research on exercise and immunity among the general public, and among college students in particular, has several clear shortcomings that also outline directions for future work. These limitations are not incidental to the topic; they shape what can currently be claimed and where the next studies should be directed.

### 8.1 “Population mismatch” in the evidence

A major issue is that the evidence relies heavily on adult, older population, or professional athlete populations, with very few original studies directly measuring immune and inflammatory markers in typical college students. This creates an awkward situation in which many claims about how college students should exercise to improve immunity are extrapolated from findings in other groups. College students have their own characteristics, such as youth, chronic academic stress, and insufficient sleep, which may result in baseline immune profiles that differ from those of middle-aged or older adults. Without data specific to this population, precise recommendations are difficult to develop.

The mismatch is more than a matter of convenience, because the groups involved differ in ways that bear directly on how exercise is translated into an immune signal. Young adults differ from older adults in immune cell composition and in the regulation of the stress response, and athletes have adapted to training volumes that a typical student body neither can nor should attempt. A recommendation derived from either group may therefore be miscalibrated in its dose as well as its direction. An intensity that is restorative for a trained runner may be excessive for a sedentary first-year student, and a training volume that is tolerable for a healthy adult may exceed what final-year students under examination pressure can sustain. The same uncertainty applies to the very outcomes of interest: suboptimal health status, as it is defined and measured in Chinese student populations, has few direct counterparts in the athlete and clinical literatures from which much of the mechanistic evidence is drawn. Until immune and inflammatory endpoints are measured in students themselves, using instruments suited to them, the field cannot know whether its recommendations are calibrated to the people they are meant to help.

### 8.2 Sample and measurement issues

Existing studies also have notable limitations in sample selection and measurement methods. In terms of samples, many surveys are confined to a single school or major, without systematic comparisons between students in sports programs and general students or across schools in different regions, resulting in insufficient comparability across institutions and regions. Regarding measurement, physical activity levels are mostly assessed through self-reported questionnaires, sedentary time is often underestimated, and objective devices remain underutilized (56, 57). There is also a lack of standardized protocols for immune and inflammatory markers: some studies measure s-IgA, others assess blood pro-inflammatory factors, and still others examine immune cell subsets. These differing indicators make direct comparisons among studies difficult and lead to fragmented conclusions.

The consequences of these choices compound one another. When exposure is self-reported and the outcome is measured with a different assay in every laboratory, any two studies differ in at least two respects at once, and it becomes impossible to attribute a disagreement to the population, the intervention, or the instrument. Sampling compounds the problem further. Studies drawn from a single campus capture its particular climate, academic calendar, and facilities, and because both physical activity and inflammation vary with season and with the timing of examinations, results collected at different points in the academic year may not be comparable even within the same institution. The absence of a shared minimum set of measures, covering both the behavioral exposure and the biological outcome, is thus a structural obstacle that no single study can overcome on its own.

### 8.3 Lack of individualized and comprehensive interventions

There is substantial individual variation in the effects of exercise on immunity; the same training regimen may elicit different responses in different individuals, yet existing research rarely considers the moderating roles of genetic background, physical fitness level, gender, and prior exercise history. In China, studies have already explored the interaction between adolescent gene polymorphisms and metabolic responses to exercise interventions, suggesting that personalized approaches are feasible (62). Another gap lies in comprehensiveness: college students often face intertwined issues such as sedentary behavior, insufficient sleep, and high stress, and relying solely on a single exercise prescription may be inadequate. Integrated intervention designs that combine exercise, sleep hygiene, and stress management are needed, and these should be tested through randomized controlled trials; such studies remain relatively scarce.

Two further difficulties attend this gap. The first is adherence. Even an optimally specified intervention has little effect if students do not complete it, and the students at greatest risk, who are typically the least active and the most stressed, are also the least likely to sustain a demanding program, so interventions risk benefiting those who need them least. The second is attribution within a multi-component design. When an intervention combines exercise, sleep, and stress management, a favorable result establishes that the package works but leaves open which element carried the effect, while a null result gives no clue which element failed. Meeting both the individualization requirement and the need for interpretable effects is a genuine design problem, and it is one reason the field still lacks the comprehensive trials that its rationale implies.

### 8.4 Future trends

Following these gaps, several trends can be anticipated. The first is objectification: wearable devices and accelerometers will enable more accurate measurement of physical activity and sedentary behavior, and a 24-h behavioral perspective that integrates sleep, sitting, and physical activity within the same day is gaining momentum. The second is individualization and precision prescription: combining genetic, metabolic, and physiological indicators to tailor appropriate types, intensities, and frequencies of exercise for different students. The third is the advancement of intervention research: randomized controlled trials with larger sample sizes, longer durations, and the incorporation of objective immune and inflammatory markers and mediating variables will be essential for determining whether exercise truly improves immunity and suboptimal health status among college students. The fourth is standardized population segmentation: establishing consistent measurement and classification criteria specifically for college students to facilitate comparability across studies. These trends converge on a single requirement, that measurement, intervention, and classification be designed together rather than in isolation, since progress on any one of them depends on the others.

### 8.5 Public health practical implications

The evidence reviewed here carries implications for how universities protect student health, and the strength of those implications varies with the evidence behind them. Because sedentary behavior, insufficient sleep, and high stress tend to co-occur and converge on the same cluster of symptoms, described in Section 3.4 as suboptimal health status, a response that targets only one of them is unlikely to succeed. It is useful to separate three tiers of recommendation, which differ in the confidence they warrant. The first tier covers measures supported by established public-health guidance for general health, for example meeting the recommended volume of moderate-intensity activity or limiting prolonged sitting, which rest on large bodies of evidence that are not specific to immune outcomes. The second tier covers measures supported specifically by immune or inflammatory outcomes in college students; this review found few such measures, and the statements advanced here are therefore deliberately limited. The third tier covers strategies that are plausible on mechanistic grounds and low in risk, but that have not yet been tested against immune or inflammatory outcomes in students, and which should be described as proposals for evaluation rather than as established practice. The five areas below are organized around what a university can act on with existing resources, and each is identified with the tier it belongs to.

Campus exercise health education (Tier 1 for physical activity itself; Tier 3 for immune outcomes). Health education should present physical activity, sleep, stress, and immune health as a connected system rather than as separate topics. Many students already know that exercise is beneficial; what is less widely understood is that the same behaviors influence inflammation and immune balance, and that the benefit depends on consistency more than on intensity. Education that frames moderate-intensity activity as the default, explains why sporadic bouts of exhausting exercise are not equivalent to regular training, and identifies low-barrier entry points such as walking, cycling, and mind–body practices is more likely to change behavior than campaigns built around performance or weight. Reviews of university-based physical activity promotion support the general principle that structured education and environmental support can shift student behavior, although these programs usually report behavioral rather than immune outcomes (58, 59, 63). Because the university years are when adult habits are consolidated, messages delivered at this stage reach students at a moment when their routines are still forming, and the value of that timing extends well beyond graduation.

Sedentary-break intervention strategies (Tier 1 for metabolic and vascular outcomes; Tier 3 for immune outcomes in students). Reducing sedentary behavior requires changes to the physical and schedule environment rather than to individual motivation alone. Because prolonged sitting is harmful even among students who meet weekly exercise targets, the most reliable interventions interrupt sitting directly: standing desks in study areas, scheduled movement breaks during long lectures and laboratory sessions, and campus planning that makes walking and cycling between buildings the natural choice rather than a special effort (6). Such measures lower the threshold for activity by removing the need to decide to exercise, which matters for students who find the idea of a formal workout session daunting. Universities can also treat class breaks as an explicit opportunity for movement and make the rationale for doing so visible, so that standing up reads as a shared norm rather than a disruption.

Coordinated sleep and stress management (Tier 1 for general health; Tier 3 for immune outcomes). Sleep and stress should be managed together with physical activity rather than in separate programs, because each influences the other and all three converge on immune balance (8). Students who are chronically stressed sleep poorly, and poor sleep reduces the energy available for activity the following day, so an intervention addressing only one element is easily undermined by the others. Practical measures include sleep-hygiene education that treats regular timing and screen use before bed as ordinary skills, accessible counseling and mindfulness services for students under examination pressure, and scheduling that avoids clustering deadlines in ways that make sleep loss unavoidable. Since exercise itself improves sleep quality and reduces anxiety and stress (18), presenting activity as a tool for managing stress, rather than as an additional obligation, may reach students who would otherwise ignore both.

Stratified exercise guidance for students of differing fitness (Tier 3). Guidance should be differentiated according to students’ fitness and health status rather than offered as a single prescription. The dose of exercise that restores immune balance in one student may be excessive for another, and the students at greatest risk, those who are sedentary, chronically stressed, or sleeping poorly, are often the least able to begin with an ambitious regimen (5). A graduated approach that starts with short, low-intensity activity and sedentary breaks, and progresses only as tolerance and habit allow, is both safer and more likely to be sustained. Tailoring is also a matter of framing: for students who rarely exercise, mind–body practices and incidental activity may be more acceptable entry points than structured training, while students already active can be guided toward balancing intensity with recovery. Universities do not need to assess every student to act on this principle; offering options across a range of intensities and making the least demanding ones visible is a practical approximation of personalization.

Implications for university health policy (Tier 3). These measures call for institutional commitments rather than isolated programs. Embedding physical activity, sedentary breaks, sleep, and stress management into university policy, through course scheduling, campus planning, health-service provision, and the training of staff who interact with students, converts what are currently voluntary and uneven efforts into a dependable baseline (64–67). Universities that collect routine data on student lifestyle and wellbeing can in addition identify which groups are most at risk and evaluate whether their measures are working, closing the loop between intervention and evidence. A system-level approach of this kind is consistent with the wider public health recognition that environments, and not only individuals, determine health behavior, and it offers universities a realistic way to address the lifestyle-related risks that this review has described.

## 9 Conclusion

This review can be summarized as follows. Physical inactivity, and in particular prolonged sitting, is associated with immune imbalance and chronic low-grade inflammation, whereas regular, appropriately dosed exercise is associated with a gradual improvement in immune balance through several pathways, including anti-inflammatory myokines, immune cell mobilization, reduced visceral fat, and better sleep and mood. Among the options considered, moderate-intensity aerobic exercise and mind–body practice have the most consistent support; high-intensity training offers performance benefits but requires careful control of dose; resistance training likely acts mainly through muscle-derived anti-inflammatory signals; and breaking up prolonged sitting is a low-cost behavior that is worth promoting in its own right. At the same time, reliable evidence that specifically targets immune markers in college students remains limited, and many of the conclusions here extend findings from other populations to students. Future work should prioritize objective measurement, individualized and integrated interventions, and stronger designs, and should read the available evidence as a connected sequence rather than as a set of competing claims. For students, the practical implication is modest and clear: consistent moderate-intensity activity, together with regular breaks from sitting, is a reasonable place to begin on the basis of its general health benefits, while its specific effect on immune outcomes in this population remains to be established.

## References

[1] Febbraio MA Pedersen BK. Exercise as a therapeutic intervention for long-lasting and chronic diseases. Cell Metab. (2026) 38:844–62. doi: 10.1016/j.cmet.2026.02.017, 41875883

[2] Pedersen BK Hoffman-Goetz L. Exercise and the immune system: regulation, integration, and adaptation. Physiol Rev. (2000) 80:1055–81. doi: 10.1152/physrev.2000.80.3.1055, 10893431

[3] Campbell JP Turner JE. Debunking the myth of exercise-induced immune suppression: redefining the impact of exercise on immunological health across the lifespan. Front Immunol. (2018) 9:648. doi: 10.3389/fimmu.2018.00648, 29713319 PMC5911985

[4] Guthold R Stevens GA Riley LM Bull FC. Worldwide trends in insufficient physical activity from 2001 to 2016: a pooled analysis of 358 population-based surveys with 1.9 million participants. Lancet Glob Health. (2018) 6:e1077–86. doi: 10.1016/S2214-109X(18)30357-7, 30193830

[5] World Health Organization. WHO Guidelines on Physical Activity and Sedentary Behaviour. Geneva: World Health Organization (2020). 33369898

[6] Castro O Bennie J Vergeer I Bosselut G Biddle SJH. How sedentary are university students? A systematic review and meta-analysis. Prev Sci. (2020) 21:332–43. doi: 10.1007/s11121-020-01093-8, 31975312

[7] Li W Zhao Z Chen D Peng Y Lu Z. Prevalence and associated factors of depression and anxiety symptoms among college students: a systematic review and meta-analysis. J Child Psychol Psychiatry. (2022) 63:1222–30. doi: 10.1111/jcpp.13606, 35297041

[8] Liu XX Wang Z Chen SJ Vitiello MV Wing YK Morin CM . Sleep health in China: status, challenges, and promotion strategies. Lancet Public Health. (2025) 10:e1055–65. doi: 10.1016/S2468-2667(25)00250-6, 41223864

[9] Furman D Campisi J Verdin E Carrera-Bastos P Targ S Franceschi C . Chronic inflammation in the etiology of disease across the life span. Nat Med. (2019) 25:1822–32. doi: 10.1038/s41591-019-0675-0, 31806905 PMC7147972

[10] Gao W Sanna M Chen YH Tsai MK Wen CP. Occupational sitting time, leisure physical activity, and all-cause and cardiovascular disease mortality. JAMA Netw Open. (2024) 7:e2350680. doi: 10.1001/jamanetworkopen.2023.50680, 38241049 PMC10799265

[11] Wang H Zhong J Hu R Fiona B Yu M Du H. Prevalence of high screen time and associated factors among students: a cross-sectional study in Zhejiang, China. BMJ Open. (2018) 8:e021493. doi: 10.1136/bmjopen-2018-021493, 29921687 PMC6009552

[12] Tong X Gao MM Zhang L Song JG Feng Y Tao Y . Chronotypes and their association with sleep quality among Chinese college students in Anhui Province: a cross-sectional study. BMJ Open. (2023) 13:e076782. doi: 10.1136/bmjopen-2023-076782 PMC10660650 37984950

[13] Wang W Jiang J Qi L Zhao F Wu J Zhu X . Relationship between mental health, sleep status, and screen time among university students during the COVID-19 pandemic: a cross-sectional study. BMJ Open. (2023) 13:e073347. doi: 10.1136/bmjopen-2023-073347, 38070905 PMC10729102

[14] Yin Y Keller J Li X Zhang T. Adherence to 24-hour movement guidelines and risk of mood disorders among Chinese university students: implications for health promotion in higher education. Int J Kinesiol High Educ. (2026) 10:1–10. doi: 10.1080/24711616.2026.2694981, 37339054

[15] Tang Z Feng S Lin J. Depression and its correlation with social support and health-promoting lifestyles among Chinese university students: a cross-sectional study. BMJ Open. (2021) 11:e044236. doi: 10.1136/bmjopen-2020-044236, 34226212 PMC8258557

[16] Reiche EMV Nunes SOV Morimoto HK. Stress, depression, the immune system, and cancer. Lancet Oncol. (2004) 5:617–25. doi: 10.1016/S1470-2045(04)01597-9, 15465465

[17] Hou H Feng X Li Y Meng Z Guo D Wang F . Suboptimal health status and psychological symptoms among Chinese college students: a perspective of predictive, preventive and personalised health. EPMA J. (2018) 9:367–77. doi: 10.1007/s13167-018-0148-4, 30538788 PMC6261912

[18] Rehman S Tanwar T Iram I Aldabbas M Veqar Z. Does regular physical activity protect sleep and mental health of university students: a systematic review. Sleep Vigil. (2024) 8:13–23. doi: 10.1007/s41782-024-00263-w

[19] Pedersen BK Febbraio MA. Muscle as an endocrine organ: focus on muscle-derived interleukin-6. Physiol Rev. (2008) 88:1379–406. doi: 10.1152/physrev.90100.2007, 18923185

[20] Steensberg A Fischer CP Keller C Møller K Pedersen BK. IL-6 enhances plasma IL-1ra, IL-10, and cortisol in humans. Am J Physiol Endocrinol Metab. (2003) 285:E433–7. doi: 10.1152/ajpendo.00074.2003, 12857678

[21] Frodermann V Rohde D Courties G Severe N Schloss MJ Amatullah H . Exercise reduces inflammatory cell production and cardiovascular inflammation via instruction of hematopoietic progenitor cells. Nat Med. (2019) 25:1761–71. doi: 10.1038/s41591-019-0633-x, 31700184 PMC6858591

[22] Peake JM Neubauer O Walsh NP Simpson RJ. Recovery of the immune system after exercise. J Appl Physiol. (2017) 122:1077–87. doi: 10.1152/japplphysiol.00622.2016, 27909225

[23] Besedovsky L Lange T Born J. Sleep and immune function. Pflugers Arch. (2012) 463:121–37. doi: 10.1007/s00424-011-1044-0, 22071480 PMC3256323

[24] Irwin MR. Why sleep is important for health: a psychoneuroimmunology perspective. Annu Rev Psychol. (2015) 66:143–72. doi: 10.1146/annurev-psych-010213-115205, 25061767 PMC4961463

[25] Irwin MR Wang M Campomayor CO Collado-Hidalgo A Cole S. Sleep deprivation and activation of morning levels of cellular and genomic markers of inflammation. Arch Intern Med. (2006) 166:1756–62. doi: 10.1001/archinte.166.16.1756, 16983055

[26] Sang D Lin K Yang Y Ran G Li B Chen C . Prolonged sleep deprivation induces a cytokine-storm-like syndrome in mammals. Cell. (2023) 186:5500–5516.e21. doi: 10.1016/j.cell.2023.10.025, 38016470

[27] Thorkildsen MS Gustad LT Mohus RM Burgess S Nilsen TIL Damas JK . Association of genetically predicted insomnia with risk of sepsis: a Mendelian randomization study. JAMA Psychiatry. (2023) 80:1061–5. doi: 10.1001/jamapsychiatry.2023.2717, 37556136 PMC10413214

[28] Segerstrom SC Miller GE. Psychological stress and the human immune system: a meta-analytic study of 30 years of inquiry. Psychol Bull. (2004) 130:601–30. doi: 10.1037/0033-2909.130.4.601, 15250815 PMC1361287

[29] Vedhara K Cox NK Wilcock GK Perks P Hunt M Anderson S . Chronic stress in elderly caregivers of dementia patients and antibody response to influenza vaccination. Lancet. (1999) 353:627–31. doi: 10.1016/S0140-6736(98)06098-X 10030328

[30] Jemmott JB Borysenko M Chapman R Borysenko JZ McClelland DC Meyer D . Academic stress, power motivation and decrease in secretion rate of salivary secretory immunoglobulin A. Lancet. (1983) 1:1400–2. doi: 10.1016/S0140-6736(83)92354-1, 6134179

[31] Campisi J Bynog P McGehee H Oakland JC Quirk S Taga C . Facebook, stress, and incidence of upper respiratory infection in undergraduate college students. Cyberpsychol Behav Soc Netw. (2012) 15:675–81. doi: 10.1089/cyber.2012.0156, 23020744

[32] Yan YX Liu YQ Li M Hu PF Guo AM Yang XH . Development and evaluation of a questionnaire for measuring suboptimal health status in urban Chinese. J Epidemiol. (2009) 19:333–41. doi: 10.2188/jea.je20080086, 19749497 PMC3924103

[33] Wang W Yan Y. Suboptimal health: a new health dimension for translational medicine. Clin Transl Med. (2012) 1:28. doi: 10.1186/2001-1326-1-28, 23369267 PMC3561061

[34] Wang W Yan Y Guo Z Hou H Garcia M Tan X . All around suboptimal health: a joint position paper of the suboptimal health study consortium and European Association for predictive, preventive and personalised medicine. EPMA J. (2021) 12:403–33. doi: 10.1007/s13167-021-00253-2, 34539937 PMC8435766

[35] Sun S Liu H Guo Z Guan Q Wang Y Wang J . Development and validation of a short-form suboptimal health status questionnaire. EPMA J. (2023) 14:601–12. doi: 10.1007/s13167-023-00339-z, 38094576 PMC10713892

[36] Xue Y Liu G Feng Y Xu M Jiang L Lin Y . Mediating effect of health consciousness in the relationship between lifestyle and suboptimal health status: a cross-sectional study involving Chinese urban residents. BMJ Open. (2020) 10:e039701. doi: 10.1136/bmjopen-2020-039701 PMC7592276 33109672

[37] Bi J Huang Y Xiao Y Cheng J Li F Wang T . Association of lifestyle factors and suboptimal health status: a cross-sectional study of Chinese students. BMJ Open. (2014) 4:e005156. doi: 10.1136/bmjopen-2014-005156, 24951109 PMC4067885

[38] Nieman DC Henson DA Austin MD Sha W. Upper respiratory tract infection is reduced in physically fit and active adults. Br J Sports Med. (2010) 45:987–92. doi: 10.1136/bjsm.2010.077875, 21041243

[39] Fedewa MV Hathaway ED Ward-Ritacco CL. Effect of exercise training on C reactive protein: a systematic review and meta-analysis of randomised and non-randomised controlled trials. Br J Sports Med. (2017) 51:670–6. doi: 10.1136/bjsports-2016-095999, 27445361

[40] McDowell SL Chaloa K Housh TJ Tharp GD Johnson GO. The effect of exercise intensity and duration on salivary immunoglobulin A. Eur J Appl Physiol Occup Physiol. (1991) 63:108–11. doi: 10.1007/BF00235178, 1748099

[41] Vossen DP McArel H Vossen JF Thompson AM. Physical activity and the common cold in undergraduate university students. Health Educ J. (2004) 63:145–57. doi: 10.1177/001789690406300205

[42] Zaitseva IP Zaitsev ON Romanov VA Semechkin NV. Immune status of students with different levels of physical activity. Russ J Immunol. (2024) 27:573–8. doi: 10.46235/1028-7221-16910-iso

[43] Rose GL Mielke GI Durr M Schaumberg MA. Effect of resistance training on chronic inflammation: a systematic review and meta-analysis. Transl Sports Med. (2021) 4:900–13. doi: 10.1002/tsm2.294

[44] Davidson RJ Kabat-Zinn J Schumacher J Rosenkranz M Muller D Santorelli SF . Alterations in brain and immune function produced by mindfulness meditation. Psychosom Med. (2003) 65:564–70. doi: 10.1097/01.PSY.0000077505.67574.E3, 12883106

[45] Oh B Bae K Lamoury G Eade T Boyle F Corless B . The effects of tai chi and qigong on immune responses: a systematic review and meta-analysis. Medicine. (2020) 7:39. doi: 10.3390/medicines7070039, 32629903 PMC7400467

[46] Ye Y Zhao F Sun S Xiong J Zheng G. The effect of Baduanjin exercise on health-related physical fitness of college students: a randomized controlled trial. Front Public Health. (2022) 10:965544. doi: 10.3389/fpubh.2022.965544, 36530684 PMC9751195

[47] Zhao Z Li T Lai H Yang R. Comparative effects of exercise modalities on depression, anxiety, and stress in university students: a systematic review and network meta-analysis of randomized controlled trials. Front Public Health. (2026) 14:1795525. doi: 10.3389/fpubh.2026.1795525, 42293647 PMC13259812

[48] Hamilton MT Hamilton DG Zderic TW. Role of low energy expenditure and sitting in obesity, metabolic syndrome, type 2 diabetes, and cardiovascular disease. Diabetes. (2007) 56:2655–67. doi: 10.2337/db07-0882, 17827399

[49] Bey L Hamilton MT. Suppression of skeletal muscle lipoprotein lipase activity during physical inactivity: a molecular reason to maintain daily low-intensity activity. J Physiol. (2003) 551:673–82. doi: 10.1113/jphysiol.2003.045591, 12815182 PMC2343229

[50] Thosar SS Bielko SL Mather KJ Johnston JD Wallace JP. Effect of prolonged sitting and breaks in sitting time on endothelial function. Med Sci Sports Exerc. (2015) 47:843–9. doi: 10.1249/MSS.0000000000000479, 25137367

[51] Pekas EJ Allen MF Park SY. Prolonged sitting and peripheral vascular function: potential mechanisms and methodological considerations. J Appl Physiol. (2023) 134:810–22. doi: 10.1152/japplphysiol.00730.2022, 36794688 PMC10042610

[52] Dunstan DW Kingwell BA Larsen R Healy GN Cerin E Hamilton MT . Breaking up prolonged sitting reduces postprandial glucose and insulin responses. Diabetes Care. (2012) 35:976–83. doi: 10.2337/dc11-1931, 22374636 PMC3329818

[53] Huang T Zheng K Li S Yang Y Kong L Zhao Y. Screen-based sedentary behaviors but not total sedentary time are associated with anxiety among college students. Front Public Health. (2022) 10:994612. doi: 10.3389/fpubh.2022.994612, 36339232 PMC9632443

[54] Petersen AMW Pedersen BK. The anti-inflammatory effect of exercise. J Appl Physiol. (2005) 98:1154–62. doi: 10.1152/japplphysiol.00164.2004, 15772055

[55] Arvidson E Dahlman AS Börjesson M Gullstrand L Jonsdottir IH. Exercise training and physiological responses to acute stress: study protocol and methodological considerations of a randomised controlled trial. BMJ Open Sport Exerc Med. (2018) 4:e000393. doi: 10.1136/bmjsem-2018-000393, 30167319 PMC6109819

[56] Panagodage-Perera NK Bullock GS Arden NK Filbay SR. Physical activity and sedentary behavior in current and former recreational and elite cricketers: a cross-sectional study. BMJ Open. (2021) 11:e052014. doi: 10.1136/bmjopen-2021-052014, 34732485 PMC8572389

[57] Guo W Key TJ Reeves GK. Accelerometer compared with questionnaire measures of physical activity in relation to body size and composition: a large cross-sectional analysis of UK Biobank. BMJ Open. (2019) 9:e024206. doi: 10.1136/bmjopen-2018-024206, 30700478 PMC6352868

[58] García-Álvarez D Faubel R. Strategies and measurement tools in physical activity promotion interventions in the university setting: a systematic review. Int J Environ Res Public Health. (2020) 17:6526. doi: 10.3390/ijerph17186526, 32911659 PMC7557565

[59] Biber D Stoepker P Davis A Ha T. College student physical activity: a systematic review. J Health Sports Kinesiol. (2022) 3:4–13. doi: 10.47544/johsk.2022.3.2.4

[60] Takahashi T Iwasaki A. Sex differences in immune responses. Science. (2021) 371:347–8. doi: 10.1126/science.abe7199, 33479140

[61] Fish EN. The X-files in immunity: sex-based differences predispose immune responses. Nat Rev Immunol. (2008) 8:737–44. doi: 10.1038/nri2394, 18728636 PMC7097214

[62] Su J Wang H Tian Y Hu H Gu W Zhang T . Impact of physical exercise intervention and PPARγ genetic polymorphisms on cardio-metabolic parameters among a Chinese youth population. BMJ Open Sport Exerc Med. (2020) 6:e000681. doi: 10.1136/bmjsem-2019-000681, 32341796 PMC7173993

[63] Lee SM Szucs LE Young E Fahrenbruch M. Using health education to address student physical activity and nutrition: evidence and implications to advance practice. J Sch Health. (2023) 93:788–98. doi: 10.1111/josh.13372, 37670599 PMC12163719

[64] Streram S Burrows T Hutchesson M. Policies and strategies for enhancing university student health behaviours: a scoping review of Australian university initiatives. Health Promot J Austr. (2026) 37:e70145. doi: 10.1002/hpja.70145, 41457146

[65] Sweeting H Thomson H Wells V Flowers P. Evolution of ‘whole institution’ approaches to improving health in tertiary education settings: a critical scoping review. Res Pap Educ. (2023) 38:661–89. doi: 10.1080/02671522.2021.1961302, 37424522 PMC7614732

[66] Dooris M Farrier A Doherty S Holt M Monk R Powell S. The UK healthy universities self-review tool: whole system impact. Health Promot Int. (2018) 33:448–57. doi: 10.1093/heapro/daw099, 28011661 PMC6160901

[67] Bachert P Wolbring L Hildebrand C Woll A Wäsche H. Analyzing mechanisms of interdisciplinary cooperation in promoting students’ health at university. BMC Public Health. (2023) 23:1911. doi: 10.1186/s12889-023-16786-2, 37789303 PMC10548763

